# *MOBP rs616147* Polymorphism and Risk of Amyotrophic Lateral Sclerosis in a Greek Population: A Case-Control Study

**DOI:** 10.3390/medicina57121337

**Published:** 2021-12-07

**Authors:** Ioannis Liampas, Vasileios Siokas, Athina-Maria Aloizou, Christos Bakirtzis, Zisis Tsouris, Anastasia Nousia, Grigorios Nasios, Dimitra Papadimitriou, Panagiotis Liakos, Dimitrios P. Bogdanos, Georgios M. Hadjigeorgiou, Efthimios Dardiotis

**Affiliations:** 1 Laboratory of Neurogenetics, Department of Neurology, University Hospital of Larissa, Faculty of Medicine, University of Thessaly, 41100 Larissa, Greece; liampasioannes@gmail.com (I.L.); bill_s1983@hotmail.com (V.S.); athena_aloi@yahoo.gr (A.-M.A.); tsouriszisis@me.com (Z.T.); hadjigeorgiou.georgios@ucy.ac.cy (G.M.H.); 2 B’ Department of Neurology, AHEPA University Hospital, Aristotle University of Thessaloniki, 54636 Thessaloniki, Greece; bakirtzischristos@yahoo.gr; 3 Department of Speech and Language Therapy, University of Ioannina, 45110 Ioannina, Greece; salogo@gmail.com (A.N.); grigoriosnasios@gmail.com (G.N.); 4 Neurological Department, Henry Dunant Hospital Center, 11526 Athens, Greece; dimipap27@yahoo.gr; 5 Laboratory of Biochemistry, Faculty of Medicine, University of Thessaly, 41100 Larissa, Greece; pliakos@med.uth.gr; 6 Department of Rheumatology and Clinical Immunology, University Hospital of Larissa, Faculty of Medicine, University of Thessaly, 41100 Larissa, Greece; bogdanos@uth.gr; 7 Department of Neurology, Medical School, University of Cyprus, Nicosia 2029, Cyprus

**Keywords:** Motor Neuron Disease (MND), Amyotrophic Lateral Sclerosis (ALS), *Myelin-associated Oligodendrocyte Basic Protein* (*MOBP*), *rs616147*

## Abstract

*Background and Objectives*: To date, only one study has investigated the association between the *rs616147* polymorphism of the *Myelin-associated Oligodendrocyte Basic Protein (MOBP)* locus and Amyotrophic Lateral Sclerosis (ALS). *Materials and Methods*: A case-control study was performed. Patients with definite sporadic ALS were prospectively and consecutively recruited from the inpatient and outpatient clinics of the Neurology Department of the General University Hospital of Larissa, Central Greece. Community based, age and sex matched healthy individuals with a free personal and family history constituted the control group. *Results*: A total of 155 patients with definite sporadic ALS and an equal number of healthy controls were genotyped. The power of our sample size was slightly above 80% and *MOBP rs616147* was determined to be in Hardy-Weinberg Equilibrium among healthy participants (*p* = 1.00). According to the univariate analysis, there was no significant relationship between *rs616147* and ALS [log-additive OR = 0.85 (0.61, 1.19), over-dominant OR = 0.73 (0.46, 1.15), recessive OR = 1.02 (0.50, 2.09), dominant OR = 0.74 (0.47, 1.16), co-dominant OR_1_ = 0.71 (0.44, 1.14) and co-dominant OR_2_ = 0.88 (0.42, 1.84). Additionally, the effect of *rs616147* on the age of ALS onset was determined insignificant using both unadjusted and adjusted (sex, site of onset) cox-proportional models. Finally, *rs616147* was not related to the site of ALS onset. *Conclusions*: Our study is the first to report the absence of an association between *MOBP rs616147* and ALS among individuals of Greek ancestry. Additional, larger nationwide and multi-ethnic studies are warranted to shed light on the connection between *rs616147* and ALS.

## 1. Introduction

Amyotrophic Lateral Sclerosis (ALS) is a progressive neurodegenerative disorder that mainly affects the upper and lower motor neurons, while about half of the patients present cognitive decline during the course of the disease [1]. The worldwide prevalence of ALS is estimated approximately between 4 and 5 patients per 100,000 individuals, whereas its incidence corresponds to about 1 to 2 new cases per 100,000 person-years [2]. ALS is more common among males and its prevalence follows an upward trend towards the 7th and 8th decades of life [2]. The mean survival of ALS patients is estimated between 2 and 4 years for most populations [2]. The only FDA-approved treatments; riluzole and edaravone, provide only small benefits regarding the median survival and clinical progress of the disease [3,4].

ALS pathology is characterized by degeneration of motor neurons in the cortex, brainstem motor nuclei and spinal anterior horns [5]. Multiple mechanisms have been associated with potential motor neurotoxicity in ALS; oxidative stress, mitochondrial dysfunction, glutamate-induced excitotoxicity, protein misfolding and aggregation, impaired axonal transportation and microglia-related mechanisms have been incriminated, but a definite conclusion for the underlying pathophysiology of ALS has not been reached [6]. Genetic and environmental parameters, as well as genetic-environmental interactions, are considered to contribute to the overall risk of the disease [7]. About 5–10% of the ALS cases are estimated to be of familial incidence, while the rest of the cases are sporadic [8]. The most important mutations associated with the familial form of the disease are related to the *C9orf72* and *SOD1* genes, as well as the FUS/TLS and TDP43 RNA binding proteins [6]. Genetic factors are considered to play an important role in the incidence of sporadic ALS too [5]. One previous Genome-wide association study (GWAS) has specifically revealed (among others) an association with a new genetic locus, *MOBP (Myelin-associated Oligodendrocyte Basic Protein)* at 3p22.1, and particularly the *rs616147* Single Nucleotide Polymorphism (SNP) [9].

*MOBP*, like myelin basic protein (MBP), is produced by oligodendrocytes and is located in the major dense line of Central Nervous System (CNS) myelin [10]. Although it is hypothesized that *MOBP* contributes to the compacting and stabilization of the myelin sheath through *MOBP*-MBP interactions, its definite function remains unclear [10]. Oligodendrocytes and myelination processes have a crucial role in several neurodegenerative diseases such as Multiple Sclerosis (MS) [11] and Alzheimer’ s Disease (AD) [12], while *MOBP* in particular has been associated with both of the aforementioned entities [13,14,15]. Furthermore, there is a relationship between Single Nucleotide Polymorphisms (SNPs) in the *MOBP* genetic locus and frontotemporal dementia (FTD) [16], a disease strongly related to ALS [1], as well as progressive supranuclear palsy (PSP), an entity of the frontotemporal lobar degeneration (FTLD) spectrum pathology [17].

Oligodendrocytes and myelination are considered important in the pathogenesis of ALS, as well. This argument is supported by changes in the composition of myelin (even demyelination) [18,19] and relevant pathological findings (including dysfunction, degeneration, defective regeneration) in grey matter oligodendrocytes of ALS subjects [20,21]. Given this background, *MOBP* could be potentially implicated in the pathogenesis of ALS. To date, only the above mentioned GWAS has identified *MOBP*, and the *rs616147* SNP (an intron variant -adenosine-guanosine replacement- of the *MOBP* gene), as a potentially ALS-associated locus [9]. Therefore, a case-control study was performed to assess the replicability of the association between *rs616147* and sporadic ALS in patients of Greek ethnicity.

## 2. Materials and Methods

A case control design was implemented in order to investigate the effect of *MOBP rs616147* on the development of ALS. The study protocol was approved by the Ethics Committee of the University of Thessaly (59295/23-01-2017) and written informed consent was obtained from all the participants. Reporting conforms with the STROBE (Strengthening the Reporting of Observational Studies in Epidemiology) reporting guidelines [22].

### 2.1. Participants and Settings

The present study involved the same participant set as two previously published articles [23,24]. Patients were prospectively and consecutively recruited from the inpatient and outpatient clinics of the Neurology Department of the General University Hospital of Larissa, Central Greece (which is affiliated with the University of Thessaly). The diagnosis of ALS was performed by a consultant neurologist according to the El Escorial criteria [25]. Community-based healthy controls were individually (1:1) matched with cases for the parameters of age (±2 years) and sex. The eligibility criteria are provided in detail below:

#### 2.1.1. Inclusion Criteria for ALS Volunteers

Age > 18 years

Greek ethnicity

Ability to provide informed consent

A diagnosis of Definite sporadic ALS based on the El Escorial criteria

#### 2.1.2. Exclusion Criteria for ALS Volunteers

Personal medical history of other neurodegenerative diseases

Family medical history of Motor Neuron Disease (MND) and FTD

#### 2.1.3. Inclusion Criteria for Healthy Volunteers

Age > 18 years

Greek ethnicity

Individual 1:1 matching for age (±2 years) and sex with the ALS individuals—enrolled from the same community as the cases

Ability to provide informed consent

No history of ALS or other neurological diseases

#### 2.1.4. Exclusion Criteria for Healthy Volunteers

Family medical history of Motor Neuron Disease (MND) and FTD

### 2.2. DNA Isolation and Genotyping

DNA was extracted using the method of salting out, which has been previously described [26,27,28]. Isolated DNA originated from peripheral blood leucocytes. Collected samples were genotyped for the *MOBP rs616147* variant using the TaqMan allele-specific discrimination assays on an ABO PRISM 7900 Sequence Detection System. Analysis of the results was performed with the SDS software (Applied Biosystems, Foster City, CA, USA). The genotype call rate was 99.03% (307/310, 152 ALS patients and 155 healthy controls).

### 2.3. Additional Data Extraction

For the ALS group, additional data were prospectively collected using standardized data abstraction In the present article emphasis was only placed on age of onset, sex and site of disease onset, categorized as bulbar, limb and mixed onset. 

### 2.4. Outcome Measures and Statistical Analysis

The primary outcome of our study was the investigation of a potential association between *MOBP rs616147* and ALS. The effect of *MOBP rs616147* on the age of ALS onset was defined as the secondary outcome. Finally, an exploratory analysis was performed to investigate for a potential association between *rs616147* and site of ALS onset.

Prior to testing the effect of *rs616147* on ALS the study quality was evaluated by testing the healthy controls for the Hardy-Weinberg equilibrium (HWE). The statistical power of our sample was estimated with the CaTS Power Calculator for Genetic Studies (Center for Statistical Genetics, University of Michigan, Ann Arbor, MI, USA) [29]. The association between *rs616147* and ALS was examined using the SNPStats software for the dominant, recessive, co-dominant, over-dominant and log-additive models of inheritance [30]. In case of more than one significant genetic models the degree of dominance (h-index) would be calculated to ‘quantify’ the mode of inheritance [31]. Cox proportional hazards models (unadjusted and adjusted for sex) were used for the examination of the effect of *rs616147* on the age of ALS onset (overall and according to the site of ALS onset). A 5% threshold was set for the definition of statistical significance. Both unadjusted and adjusted effect sizes (Odds Ratio -OR-, Hazard Ratio -HR-) and their precision (95% Confidence Interval -95% CI-) are presented. Cox proportional hazards regression was carried out with the IBM SPSS Statistics Software version 23.0 for Windows (SPSS Inc., Chicago, IL, USA). Finally, the association between *rs616147* and site of ALS onset (limb or bulbar) was examined using the SNPStats software, for the dominant, recessive, co-dominant, over-dominant and log-additive models of inheritance.

## 3. Results

All of the patients and controls that were invited to participate in the study responded positively and completed the study processes. A total of 155 patients with definite sporadic ALS and an equal number of age (±2 years) and sex matched healthy controls were recruited. As noted above, the genotype call rate was 99.03%, with a total of 152 ALS patients and 155 healthy controls ultimately having available genetic data. The power of our sample size was slightly above 80% to find a significant association (*p* < 0.05) between *MOBP rs616147* and ALS, given a minor (A) allele frequency of 32% [32], a disease prevalence of 5/100,000 [2], and an estimated relative risk of 1.60. Patient characteristics are presented in Table 1. *MOBP rs616147* was determined to be in HWE among healthy participants (*p* = 1.00). Allelic and genotypic frequencies are provided in Table 2. The minor allele (A) frequencies were 29% and 33% for the cases and controls, respectively.

According to the univariate analysis, there was no significant relationship between *MOBP rs616147* and ALS (primary outcome measure) with respect to every mode of inheritance; log-additive OR = 0.85 (0.61, 1.19), over-dominant OR = 0.73 (0.46, 1.15), recessive OR = 1.02 (0.50, 2.09), dominant OR = 0.74 (0.47, 1.16), co-dominant OR_1_ = 0.71 (0.44, 1.14) and co-dominant OR_2_ = 0.88 (0.42, 1.84) (Table 3).

Additionally, the effect of *rs616147* on the age of ALS onset was investigated (Table 4). Both crude (G/G vs. G/A; HR = 1.12 (0.80, 1.59), G/G vs. A/A; HR = 0.91 (0.54, 1.54)) and sex-adjusted (G/G vs. G/A; HR = 1.11 (0.79, 1.56), G/G vs. A/A; HR = 0.94 (0.55, 1.60)) cox-proportional hazards models provided evidence indicative of no association between *rs616147* and age of ALS onset (the effect of sex on the age of ALS onset was determined insignificant; HR = 1.37 (0.96, 1.84)). Subgroup analyses based on the site of onset, reproduced the insignificant associations, both when unadjusted and adjusted for sex (the effect of sex on the age of ALS onset was determined insignificant regarding the limb onset ALS; HR; 1.44, (0.85, 2.44), but significant regarding the bulbar onset ALS; HR = 3.43 (1.29, 9.09), with male sex presenting later onset of the disease) (Table 5).

Finally, no association was found between *rs616147* and site of ALS onset (Table 6). Limb onset (vs. bulbar and mixed onset) and bulbar onset (vs. limb and mixed) were separately analysed.

## 4. Discussion

The present case-control study investigated the effect of *MOBP rs616147* on the development of ALS, as well as the age and site of ALS onset. Results were compatible with the absence of an underlying connection, regarding all outcome measures. Our findings come in contradiction with the findings of the only other study that examined this association, a previous very large GWAS [9]. The aforementioned study involved data from a great number of ALS cohorts originating from Western countries, but a Greek cohort was not included. Apart from the *MOBP rs616147*—ALS direct association, a dose-dependent pharmacogenetic interaction has been recently revealed between the A allele of *rs616147* and creatine administration to patients with ALS [33].

The functions of *MOBP* are not yet completely clarified (the functional significance of the *rs616147* polymorphism as well), but it appears to participate in myelin compaction and stabilization, through interactions with a structurally similar protein, MBP [10,34,35]. Oligodengroglial dysfunction and myelination disorders are crucial in ALS [36]. Grey matter oligodendrocytes present substantial degeneration in ALS animal models, while precursor cells fail to differentiate and compensate for the losses, ultimately leading to incomplete recovery. The degeneration of oligodendrocytes subsequently causes myelin abnormalities, such as immature myelin sheaths and demyelinated axons, and, finally, leads to axonal degeneration. These pathological findings support the notion that myelination processes (as well as myelination-related loci such as *MOBP*) are potentially important for ALS. On the other hand, *rs616147*, which is an intron variant of the *MOBP* gene, may not directly affect the function of *MOBP* and by extension myelination, rendering the myelination abnormalities among ALS patients irrelevant.

The association of *MOBP* with other neurodegenerative diseases is well-established. To date, autoreactivity against *MOBP* has been detected among individuals with MS [13,15] and *MOBP* immunoreactivity has been detected in the core of Lewy Bodies (LBs) among patients with Parkinson’ s disease and dementia with LBs [37,38]. *MOBP* SNPs have been associated with Apolipoprotein-E e4 positive AD [14], FTD (and the severity of white matter degeneration [16,39]), PSP [17,40,41,42,43,44], Corticobasal Degeneration [43,44], while decreased expression of *MOBP* was revealed in familial Globular Glial Tauopathy [45] and differential DNA methylation of *MOBP* was shown in Multiple System Atrophy [46]. The implication of *MOBP* in all these neurodegenerative disorders makes it possible that it is also involved in the pathophysiology of ALS.

The study, nevertheless, failed to demonstrate the existence of an association. The present study was the first to examine this SNP among individuals of Greek ethnicity with sporadic ALS. Therefore, the replicability of our results cannot be tested. At this stage, it is appropriate to recognize that our study has some limitations, which may be accountable for the non-significant results. First, the power of our study was slightly over 80%, which means that there is an almost 20% possibility that we failed to detect a truly significant association. Additionally, both primary and secondary analyses were not adjusted for several potential protective or risk-conferring factors, including both genetic and environmental ones (such as pesticide and metal exposure) [8,47]. Therefore, our results may be significantly affected by the latent effect of an uncontrolled parameter. Moreover, both patients and healthy controls (matched for sex and age ±2 years, selected from the same community) were recruited from a specific geographical area located in Central Greece. The selection of this sample achieved several advantages by possibly matching for several undetermined exposures to a variety of environmental factors (e.g., soil and water metal concentrations) but might have induced an overmatching bias when it comes to genetic parameters. Finally, the conduction of a GWAS is more appropriate than the investigation of a single locus for distinguishing disease vulnerability genes in sporadic maladies. Thus, ideally, a large-scale GWAS with Greek ALS patients should be conducted to evaluate the association of *rs616147* with ALS and individuals of Greek ethnicity.

## 5. Conclusions

It is warranted that additional studies are performed to shed light to the relationship between *MOBP rs616147* and ALS. Larger, nationwide studies will better capture the underlying connection among individuals of Greek ancestry, while multi-ethnic studies will reveal the differences among different populations. Finally, it is of substantial importance that future research addresses the latent effect of significant genetic and environmental exposures, that are already considered to confer susceptibility to ALS (at least lead exposure and possibly pesticide exposure [47]). At the same time, the comprehensive collection of additional clinical information, as well as the complete and transparent reporting [48,49], will elucidate the potential connection of *rs616147* with the treatment-related and prognostic parameters.

## Figures and Tables

**Table 1:** Characteristics of the ALS participants.

Assessed Parameters	Measurements
Age (mean years ± SD, median (IQR))	63.74 ± 11.30, 65 (57, 72)
Sex (Female/Male)	77/78
Site of Onset (Bulbar/Spinal/Mixed)	50/97/8

ALS: Amyotrophic Lateral Sclerosis; SD: standard deviation; IQR: interquartile range.

**Table 2:** Allelic and genotypic frequencies for *MOBP rs616147* in ALS patients and healthy controls.

	ALS Patients (%)	Healthy Controls (%)	Total Participants (%)
Genotypes	152	155	307
A/A	17 (11%)	17 (11%)	34 (11%)
G/A	55 (36%)	68 (44%)	123 (40%)
G/G	80 (53%)	70 (45%)	150 (49%)
Alleles	304	310	614
A	89 (29%)	102 (33%)	191 (31%)
G	215 (71%)	208 (67%)	423 (69%)

*MOBP*: *Myelin-associated Oligodendrocyte Basic Protein*; ALS: Amyotrophic Lateral Sclerosis.

**Table 3:** Single locus association of *MOBP rs616147* with ALS.

Mode of Inheritance	Genotype	Odds Ratio (95% Confidence Interval)	*p*-Value
Co-dominant	G/G	1.00	0.37
	G/A	0.71 (0.44, 1.14)	NA
	A/A	0.88 (0.42, 1.84)	NA
Dominant	G/G	1.00	0.19
	G/A-AA	0.74 (0.47, 1.16)	NA
Recessive	G/A-G/G	1.00	0.95
	A/A	1.02 (0.50, 2.09)	NA
Over-dominant	G/G-A/A	1.00	0.17
	G/A	0.73 (0.46, 1.15)	NA
Log-additive	-	0.85 (0.61, 1.19)	0.35

*MOBP*: *Myelin-associated Oligodendrocyte Basic Protein*; ALS: Amyotrophic Lateral Sclerosis; NA: not applicable.

**Table 4:** Single locus association of *MOBP rs616147* with the age of ALS onset (crude and adjusted for sex).

Genotype	Univariate		Multivariate	
	Hazard Ratio (95% CI)	*p*-Value	Hazard Ratio (95% CI)	*p*-Value
G/G	1.00	NA	1.00	NA
G/A	1.12 (0.80, 1.59)	0.51	1.11 (0.79, 1.56)	0.56
A/A	0.91 (0.54, 1.54)	0.71	0.94 (0.55, 1.60)	0.82

*MOBP*: *Myelin-associated Oligodendrocyte Basic Protein*; ALS: Amyotrophic Lateral Sclerosis; CI: confidence interval; NA: not applicable.

**Table 5:** Single locus association of *MOBP rs616147* with the age of ALS onset (crude and adjusted for sex) according to the site of onset (bulbar of limb).

Genotype	Bulbar Onset				Limb Onset			
	Univariate		Multivariate		Univariate		Multivariate	
	HR (95% CI)	*p*-Value	HR (95% CI)	*p*-Value	HR (95%CI)	*p*-Value	HR (95% CI)	*p*-Value
G/G	1.00	NA	1.00	NA	1.00	NA	1.00	NA
G/A	1.46 (0.68, 3.14)	0.34	1.67 (0.77, 3.65)	0.20	1.57 (0.91, 2.70)	0.11	1.57 (0.91, 2.72)	0.10
A/A	1.10 (0.31, 3.87)	0.88	1.29 (0.36, 4.60)	0.69	1.77 (0.77, 4.10)	0.18	1.77 (0.76, 4.09)	0.18

*MOBP*: *Myelin-associated Oligodendrocyte Basic Protein*; ALS: Amyotrophic Lateral Sclerosis; HR: Hazard Ratio; CI: Confidence Interval; NA: not applicable.

**Table 6:** Single locus association of *MOBP rs616147* with site of ALS onset.

Limb onset vs. Bulbar or mixed onset	**Mode of Inheritance**	**Genotype**	**Odds Ratio (95% Confidence Interval)**	***p*** **-** **Value**
	Co-dominant	G/G	1.00	0.72
		G/A	0.77 (0.41, 1.44)	NA
		A/A	0.90 (0.34, 2.39)	NA
	Dominant	G/G	1.00	0.45
		G/A-AA	0.80 (0.44, 1.43)	NA
	Recessive	G/A-G/G	1.00	0.98
		A/A	1.01 (0.40, 2.58)	NA
	Over-dominant	G/G-A/A	1.00	0.43
		G/A	0.79 (0.43, 1.43)	NA
	Log-additive	-	0.88 (0.57, 1.37)	0.58
Bulbar onset vs. Limb or mixed onset	**Mode of Inheritance**	**Genotype**	**Odds Ratio (95% Confidence Interval)**	***p*** **-** **Value**
	Co-dominant	G/G	1.00	0.88
		G/A	0.84 (0.37, 1.87)	NA
		A/A	0.77 (0.20, 2.96)	NA
	Dominant	G/G	1.00	0.62
		G/A-AA	0.82 (0.38, 1.76)	NA
	Recessive	G/A-G/G	1.00	0.79
		A/A	0.84 (0.23, 3.05)	NA
	Over-dominant	G/G-A/A	1.00	0.74
		G/A	0.88 (0.40, 1.90)	NA
	Log-additive	-	0.86 (0.48, 1.55)	0.62

*MOBP*: *Myelin-associated Oligodendrocyte Basic Protein*; ALS: Amyotrophic Lateral Sclerosis; NA: not applicable.

## Data Availability

The data that support the findings of this study are available from the corresponding author E.D., upon reasonable request.
